# Acupuncture effect on dumping syndrome in esophagus cancer patients with feeding jejunostomy: A study protocol for a single blind randomized control trial

**DOI:** 10.1097/MD.0000000000033895

**Published:** 2023-06-09

**Authors:** Peter Karl Mayer, Pei-Yu Kao, Yu-Chen Lee, Yi-Fang Liao, Wen-Chao Ho, Eyal Ben-Arie

**Affiliations:** a International Master Program in Acupuncture, College of Chinese Medicine, China Medical University, Taichung, Taiwan; b Department of Chinese Medicine, China Medical University Hospital, Taichung, Taiwan; c Institute of Traditional Medicine, School of Medicine, National Yang-Ming University, Taipei, Taiwan; d Surgical Intensive Care Unit, China Medical University Hospital, Taichung, Taiwan; e Division of Thoracic Surgery, Department of Surgery, China Medical University Hospital, Taichung, Taiwan; f Department of Acupuncture, China Medical University Hospital, Taichung, Taiwan; g Chinese Medicine Research Center, China Medical University, Taichung, Taiwan; h Graduate Institute of Acupuncture Science, College of Chinese Medicine, China Medical University, Taichung, Taiwan; i Department of Public Health, China Medical University, Taichung, Taiwan.

**Keywords:** acupuncture, digestion, dumping syndrome, esophagus cancer, feeding jejunostomy, malnourishment, weight loss

## Abstract

Esophagus cancer patients are at risk for malnourishment. Feeding jejunostomy is used in advanced esophagus cancer patients in order to support and supplement the patients’ nutrition needs. In dumping syndrome, the food is rapidly introduced into the intestine at a rate that is faster than normal, it is associated with both digestive system and vasoactive symptoms. Dumping syndrome has an association with both esophagus cancer patients and feeding jejunostomy. In the mid and long term, dumping syndrome is an important issue that contributes to the risk of malnourishment in advanced esophagus cancer patients. In recent studies, acupuncture was effective in regulating digestive symptoms. Acupuncture is considered to be a safe intervention, that was previously shown to be effective in treating digestive-related symptoms.

**Methods:**

Sixty advanced esophageal cancer patients post-feeding jejunostomy will be divided into 2 equal groups, an intervention group (n = 30) and a control group (n = 30). Patients in the intervention group will receive acupuncture using the following acupoints: ST36 (Zusanli), ST37 (Shangjuxu), ST39 (Xiajuxu), PC6 (Neiguan), LI4 (Hegu), and Liv 3 (Taichung). Patients in the control group will receive shallow acupuncture on 12 non-acupoints (sham points), 1 cm from the above mention points. Patients and assessors will be blind to trial allocation. Both groups will receive acupuncture twice a week for 6 weeks. The main outcome measurements are: body weight, BMI, Sigstad’s score, and the Arts’ dumping questionnaire.

**Discussion:**

There are no previous studies that have examined the use of acupuncture on patients with dumping syndrome. This single-blind randomized control trial will investigate the effect of acupuncture on dumping syndrome in advanced esophagus cancer patients with feeding jejunostomy. The results will determine if verum acupuncture can affect dumping syndrome and prevent weight loss.

## 1. Introduction

Esophagus cancer’s new cases in 2020 were 604,100, the 10th most common cancer in the world. Esophagus cancer also had an astonishing 544,076 new deaths in 2020, which represents 5.5 percent of all cancer mortality.^[1,2]^ Based on the current trend the forecast for the burden of esophagus cancer is expecting an increase to 739,666 new cases and 723,466 deaths in 2030, and 987,723 new cases and 914,304 deaths in 2040.^[2]^ Not long ago, the 5 years survival for esophageal cancer was 21.7% (United States data from 2011–2019).^[3]^ Esophagus cancer patients often suffer from progressive dysphagia due to the tumor and its effect on the integrity and function of the esophagus, which leads to serious weight loss and possible malnourishment.^[4]^ A study on 154 French hospital wards found that 60.2% of the patients with esophagus and/or stomach cancer were malnourished.^[5]^ In a study including 1482 esophageal cancer patients existing prevalence of malnutrition was around 50% of the patients, when measured by the NRS-2002.^[6]^ Advanced esophagus cancer patients have a limited oral feeding ability and their nutrition support is often provided through a jejunostomy tube feeding. Especially at the time of preoperative chemotherapy and radiotherapy sessions.^[7,8]^ In clinical observation of patients after jejunostomy, the presence of dumping syndrome was observed, due to the faster speed at which the food enters the intestine through the jejunostomy tube.^[9]^ The prevalence of dumping syndrome is considered high among esophagus cancer patients, with a prevalence ranging between 20-50% of the patients.^[10,11]^ Dumping syndrome is a condition in which the food is rapidly introduced into the intestine at a rate that is faster than normal, this usually occurs due to anatomical and physiological changes in the digestive system. Dumping syndrome’s main symptom is diarrhea, other symptoms contain abdominal cramps, nausea, and vasoactive symptoms like tachycardia, palpitations, fatigue, a desire to lie down following eating, pallor, diaphoresis, lightheadedness, hypotension, headache, and syncope.^[12,13]^ This often occurs 1 hour after a meal although late dumping can occur 1 to 3 hours after a meal.^[12,14]^ After the food rapidly enters the intestine a large number of hormones are secreted, such as neurostatin, vasoactive intestinal peptide, incretins, insulin, and glucagon which cause the vasomotor symptoms to appear.^[14]^ Dumping syndrome is also associated with weight loss.^[15,16]^ This is particularly important for esophagus cancer patients due to their initial high risk of malnourishment.^[5]^ The management of dumping syndrome in Western medicine is mainly based on dietary adjustments.^[16]^

Acupuncture is a treatment that was previously shown to reduce diarrhea for irritable bowel syndrome patients^[17,18]^ along with a reduction in gastroparesis syndrome.^[19,20]^ Acupuncture was also shown the ability to regulate gastrointestinal mobility through different physiological pathways.^[21]^ Acupuncture decreased the fasting glucagon-like peptide-1 levels in an animal model^[22]^ and also showed the ability to decrease insulin and glucagon levels.^[23]^ In oncological patients, acupuncture reduced chemotherapy-related abdominal pain, nausea, vomiting, and diarrhea with good safety.^[24–26]^ We have chosen to compare acupuncture to shallow needling on sham non-acupoint in order to generate a reliable placebo effect that resembles acupuncture intervention processes.

There are no previous studies that have examined the direct use of acupuncture on dumping syndrome. The goal of this single-blind randomized control trial is to investigate the effect of acupuncture on dumping syndrome in esophagus cancer patients with jejunostomy tube feeding. We hypothesize that acupuncture will be superior to sham acupuncture in regulating dumping syndrome.

## 2. Methods

### 2.1. Study design and settings

**Figure 1.**
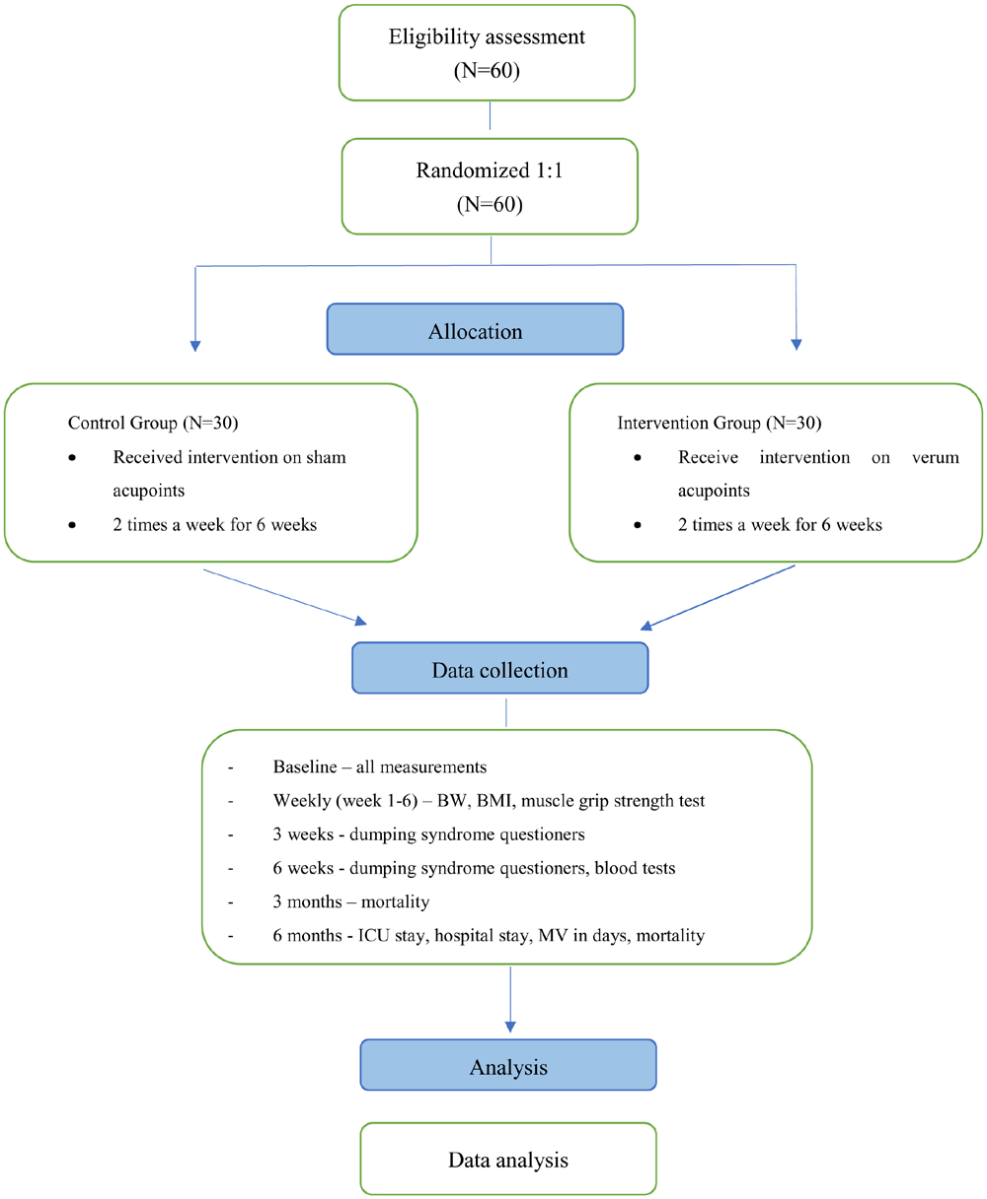
Study flow chart. The study flow chart describes the study processes. BW = body weight, BMI = body mass index, MV = mechanical ventilation, ICU = intensive care unit.

This single-blind randomized control trial will be implemented in the Department of thoracic surgery of China medical university hospital (CMUH), Taichung City, Taiwan. The study will be initiated on February 2023 and will be concluded until January 2025. This single center study will use a 1:1 ratio parallel group allocation. Informed consent will be obtained prior to the patient’s enrollment. The 2 groups are an intervention group (INT) and a control group (CON). The study was approved by the institutional review board (IRB) of CMUH (approval no. CMUH111-REC3-004) and a study protocol registration online on https://clinicaltrials.gov/ct2/show/NCT05801666 (registration no. NCT05801666). The study aims to examine the effect of acupuncture on preventing and treating dumping syndrome in esophagus cancer patients after feeding jejunostomy surgery. The study will also measure the effect of acupuncture on preventing malnutrition using body weight and body mass index (BMI) (see Fig. 1). In this study, both the patients and the assessors of outcome measurements will be blinded to the group’s allocation.

### 2.2. Participants

Sixty esophagus cancer patients after feeding jejunostomy from CMUH from the department of chest surgery will be included in this study. The patients will be randomly divided equally into 30 patients in the intervention or the control group after meeting the study’s inclusion criteria and obtaining informed consent from the patients (or a legal guardian).

### 2.3. Inclusion criteria

Age: 20 to 80-year-old

Advanced esophageal cancer

Post feeding jejunostomy

Plan of Concurrent Chemoradiation Therapy for esophageal cancer

### 2.4. Exclusion criteria

Acute infection

Unstable vital signs

Other medical conditions which would affect nutrition status

### 2.5. Recruitment strategies

During a patient’s consultation with a doctor, the doctor will inform the relevant patients about a study taking place. Study personnel will explain the pros and cons of the study in case the patient intends to enroll in the study. The study personnel will arrange meetings with the doctors in the department of thoracic surgery in order to increase the department doctor’s awareness for the study inclusion criteria.

### 2.6. Informed consent

Once a patient meets the inclusion criteria, the patient or the patient’s legal guardian will be asked for interest in joining the study. Study personnel will explain the benefits and risks of the study prior the signing informed consent. After signing the informed consent, the patient has the right to ask for study withdrawal at any time. If the patient agrees, his/her data will be still used for the study data analysis.

### 2.7. Randomization and allocation concealment

For study randomization, a 1:1 simple computer-based random number table without stratification was generated via IBM SPSS statistics version 22 (SPSS Inc, Chicago, IL). The 2 groups’ random sequentially number along with correlated acupuncture/sham points picture and name was placed inside 60 nontransparent, sealed envelopes by a study personnel B-AE. At the time of intervention, a study research assistant will deliver a random envelope to the acupuncture doctor. The acupuncture doctor will attach the patient’s information to the envelope and will provide the intervention according to the acupuncture/sham points in the envelope.

### 2.8. Interventions

A sum of 60 patients from the China medical university will be divided into 2 groups. In both the INT group and CON group, A qualified acupuncture doctor with at least 2 years of clinical experience will provide the intervention sessions on both groups. The acupuncture frequency will be 2 times a week for 6 weeks in both groups. Both groups will also receive routine Western medicine treatments.

### 2.9. Intervention group (INT)

**Figure 2.**
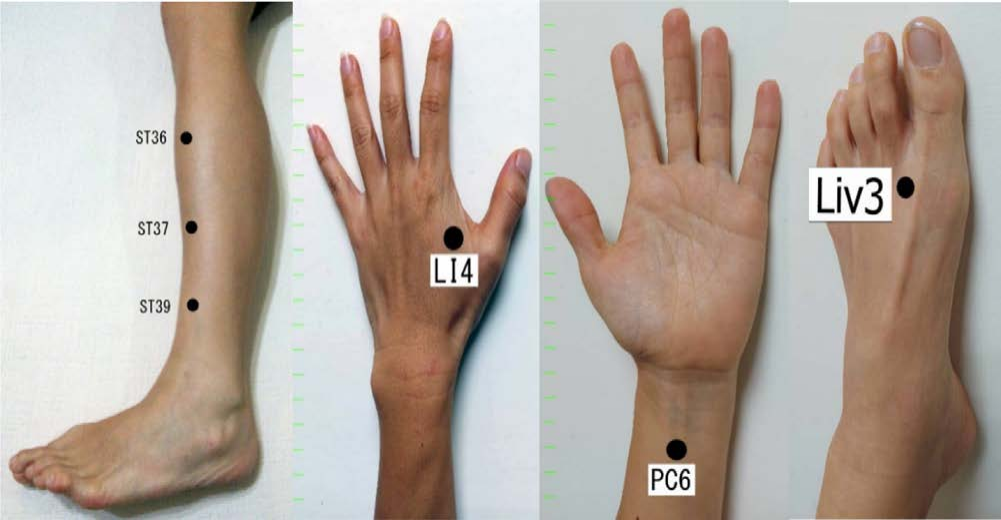
Acupoints used in the intervention group. Intervention group acupoints: ST36 (Zusanli), ST37 (Shangjuxu), ST39 (Xiajuxu), PC6 (Neiguan), and LI4 (Hegu) liv 3 (Taichung).

In the intervention group, the patients will receive traditional Chinese medicine style acupuncture at the following points: ST36 (Zusanli), ST37 (Shangjuxu), ST39 (Xiajuxu), PC6 (Neiguan), and LI4 (Hegu) Liv 3 (Taichung) (Fig. 2). The points will be applied bilaterally. The effectiveness of those points in treating digestion (with the exception of Liv 3 [Taichung]) was shown in our previous research.^[27]^ The treatment will take place 2 times a week for 6 weeks. Totally 12 needles will be used in each intervention. Acupuncture sterile needles manufactured by “Yu Kuang” 40mm with 30G will be used for this study (Fig. 2).

As part of the acupuncture procedure, the acupuncturist will disinfect the acupuncture points with 70% alcohol and will insert the needles perpendicularly into the muscle layer at an approximate depth of 10 to 30 mm. The doctor will manipulate the needles upward and downward and will rotate the needles 180° in both directions in a search for the “De Chi” phenomenon. During the intervention, the patient will be positioned in a supine position. After 30 minutes the doctor will withdraw the needles.

### 2.10. Control group (CON)

**Figure 3.**
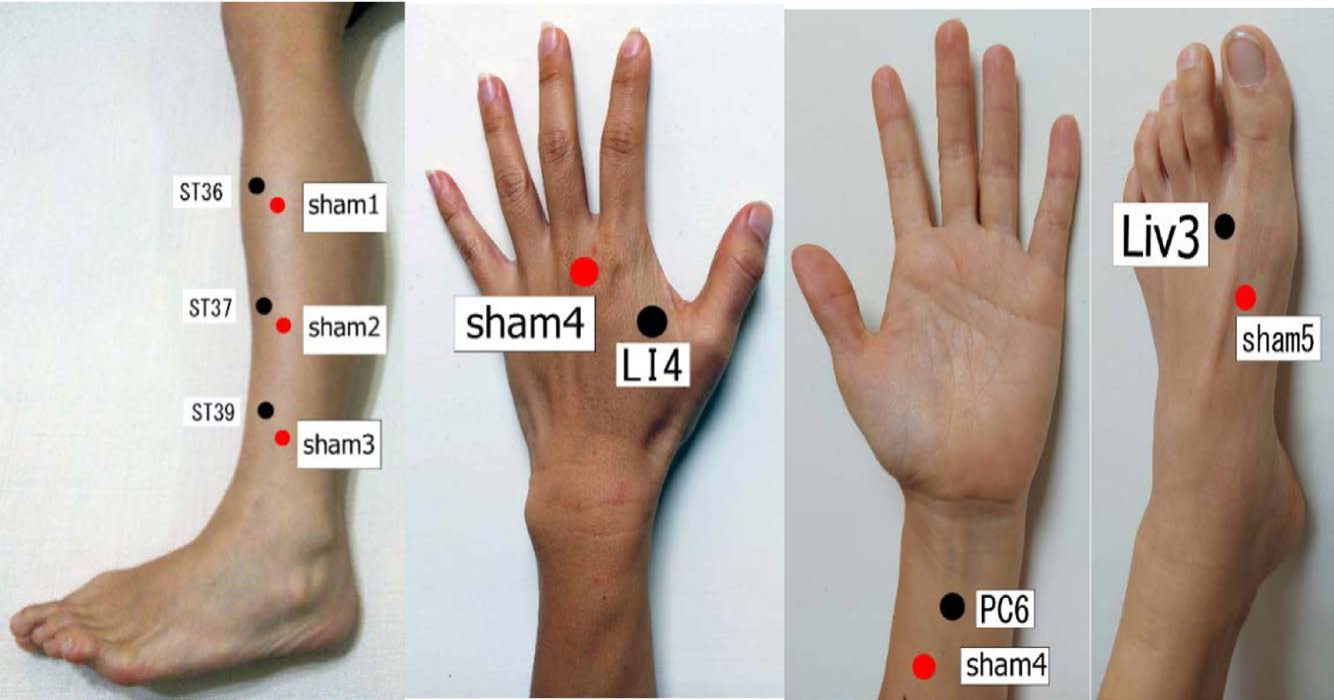
Sham acupoints used in the control group. The red dots represent the sham points used in the control group.

The patients in the control group will receive bilateral shallow acupuncture (12 needles) on the sham points that are located 1 cm lateral/medial and 1 cm distal/proximal to the following acupoints: ST36 (Zusanli), ST37 (Shangjuxu), ST39 (Xiajuxu), PC6 (Neiguan), and LI4 (Hegu) liv 3 (Taichung) (Fig. 3). Those points are in a location of 1 cm lateral/medial and 1 cm distal/proximal to those points and are not known as acupuncture points. The needle insertion will be a perpendicular shallow insertion into the skin tissue level. The acupuncture doctor will use the needle with tube insertion for needle insertion and will leave the needle in the depth of the tube insertion (approximately 4 mm depth into the skin) without needle manipulation. The needles will be retained for 30 minutes. In addition to the sham points, the patients in this group will also receive routine Western medicine treatment as per each patient’s needs. The treatment frequency and skin disinfection will be identical to the treatment group.

### 2.11. Drug treatment

All patients will receive drug treatments as prescribed by their doctor. This study will not alter any drug intervention.

### 2.12. Outcome measures

Main outcome measurements are: body weight, BMI (will be measured weekly) dumping syndrome questioners: the Sigstad’s score in order to determine the diagnosis of dumping syndrome and the Arts’ dumping questionnaire in order to assess early or late dumping syndrome (will be measured on the baseline, week 3, week 6) (see Figure S1, Supplemental Digital Content, http://links.lww.com/MD/J34 for the Sigstad’s score and Figure S2, Supplemental Digital Content, http://links.lww.com/MD/J35 for the Arts’ dumping questionnaire).

**Figure 4.**
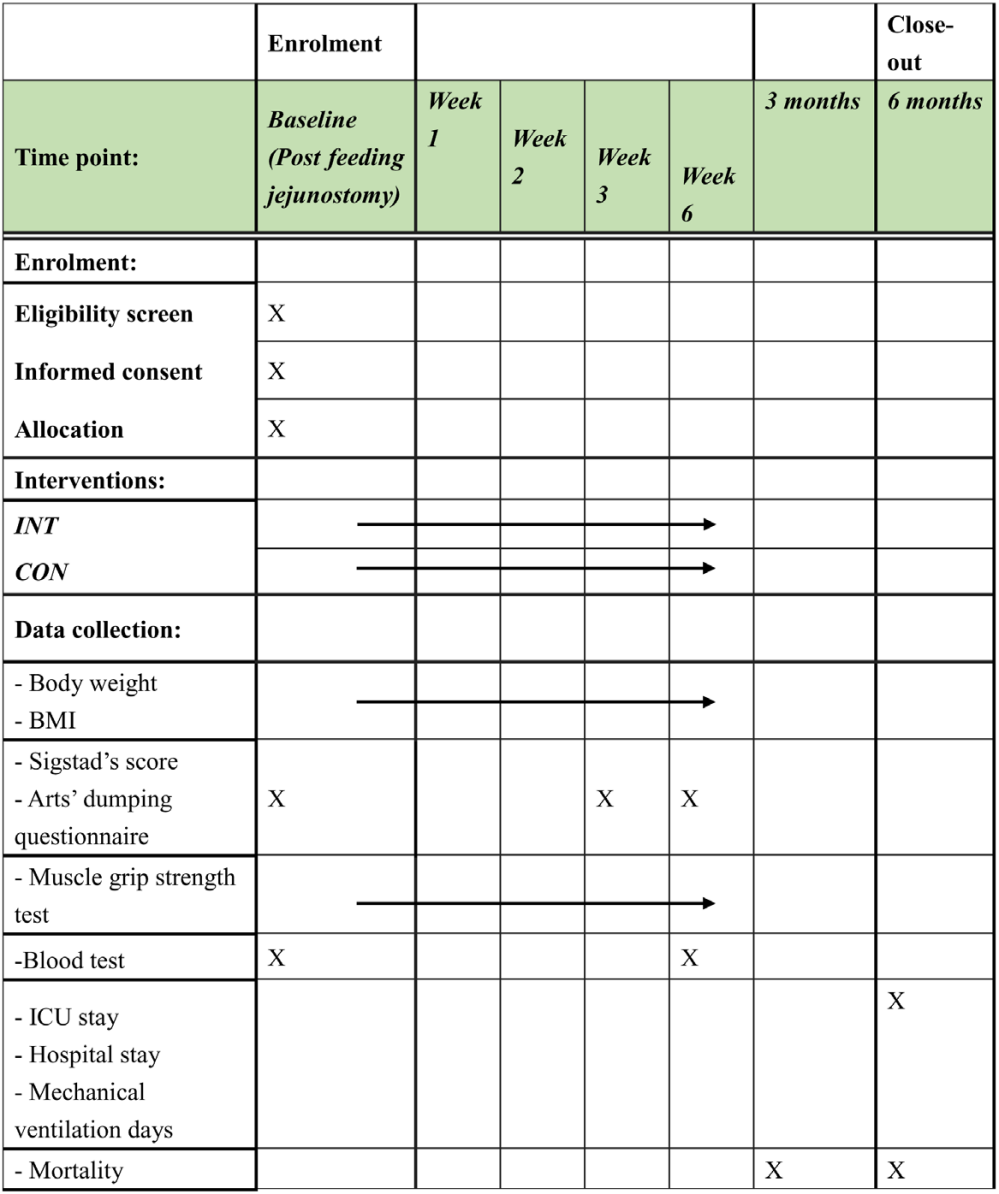
Study schedule. The study schedule describes which intervention and measurements will be measured in which time period. BMI = body mass index, ICU = intensive care unit.

The secondary outcomes are: muscle grip strength test (measured weekly in kg) (by electronic hand grip test model EH101 manufactured by “CAMRY,” China), blood test: Complete Blood Count, kidney and electrolytes (measured as adjuvant adjuvant concurrent chemoradiation therapy schedule), pre-albumin, albumin level, glucose blood levels, inflammation data including Erythrocyte Sedimentation Rate and C-reactive protein (measured at baseline and on week 6). Total Intensive care unit stay, total hospital stay, and total mechanical ventilation in days will be measured 6 months after inclusion. Mortality will be measured at 3 months, 6 months. Medical charts and drug use will also be measured (Fig. 4). All measurements will be measured by nurses and doctors in the department that are blind to the study group allocation.

### 2.13. Adverse events

Any acupuncture-related adverse events including minor bleeding, hematoma, infections, alcohol allergy, etc. will be recorded in detail and reported in the study findings. In the event of any serious adverse events the group allocation will be revealed to the patient and the assessors and the patient will receive adequate treatment. The primary investigator and IRB committee will be informed immediately, the IRB committee will determine if the study requires modification or needed to be stopped.

### 2.14. Sample size calculation

Since no previous similar study was conducted, the sample size was determined via the G*Power version 3.1.9.4 software calculation, based on a large effect for a two-tailed *t* test. The calculation showed that a total of 26 patients were needed. However, to account for a 15% patient drop-out expected frequency. For 80% power with an effect size of 0.8, a total of 60 patients were required, with 30 patients in each group.

### 2.15. Data management

To ensure the accuracy and reliability of data, patient data will be uploaded to the CMU hospital electronic database, along with manually written reports on measurements that are not usually uploaded to the system. The manually written report will be stored in a secure location in the office of the principal investigator and is scheduled to be destroyed 5 years post trial completion. The principal investigator will also arrange meetings with study staff to ensure the patients’ enrollment and interventions are according to the study protocol.

### 2.16. Statistical analysis

The statistical analysis for all measurements will analyze changes from baseline and changes over time. Categorical data will be presented as a percentage (n %), while continuous data will be presented as mean ± standard deviation. For changes from baseline the paired *t* test will be used. The independent *t* test or the Mann–Whitney *U* test will analyze continuous variables depending on applicability, and the Chi-square test or Fisher’s exact test (as appropriate) will be used for categorical variables. For repeat measurements data the repeat measures ANOVA will be used. The IBM SPSS Statistics version 22.0 (SPSS Inc, Chicago, IL) will be used for data analysis, and significance will be determined at a *P* value < .05. If applicable, a subgroup analysis will be done on tumor stage, age, BMI, dumping syndrome status at baseline and dumping syndrome type. If there are any missing data, they will be imputed using multiple imputations (if applicable).

### 2.17. Dissemination

After study completion, the study results will be published in a medical journal.

## 3. Discussion

Dumping syndrome is often a side effect of digestive system physical alterations such as surgeries. The most common are esophageal, gastric, or bariatric surgeries.^[28]^ The rapid movement of hyperosmolar chyme leads to gastrointestinal and vasomotor symptoms.^[14]^ Dumping syndrome can lead to significant weight loss that can be resulted in hospitalization for hypoglycemia. It is especially an issue for advanced esophagus cancer patients as their high risk of malnourishment and can contribute to the patient’s extremely low 5 years survival rate.^[5,29]^

Although there is no previous study that investigated the effect of acupuncture on dumping syndrome, acupuncture showed great potential in alleviating digestive system symptoms. Acupuncture was found to be superior to conventional treatments for diarrhea in a meta-analysis of 17 studies on irritable bowel syndrome patients.^[30]^ The acupoint PC6 (Neiguan) effect on nausea was shown to be beneficial in a Cochrane meta-analysis of 59 studies, although the level of evidence was low.^[31]^ A meta-analysis of 20 studies found acupuncture to be superior to placebo and usual care in postoperative nausea and vomiting.^[32]^ Acupoint stimulation is also effective for postoperative nausea and vomiting after general anesthesia.^[33]^ For postoperative gastroparesis syndrome, a meta-analysis found that both acupuncture and acupuncture combined with medication showed a significant effect compared to the control, however, the level of evidence was low.^[19]^ In a clinical trial investigating acupuncture for patients after postoperative laparoscopic common bile duct exploration, the acupoints ST36 (Zusanli), ST37 (Shangjuxu), and ST39 (Xiajuxu) with early Enteral Nutrition (EN) significantly improve the patient’s outcomes in digestive symptoms, had fewer complications, and a shorter hospital stay.^[34]^ In our previous clinical trial, acupuncture on the digestion specific points reduce postoperative feeding intolerance and reduce the use of the prokinetic drug in critically ill oral cancer patients.^[27]^ Acupuncture showed to be as effective as oral administration of Mosapride for patients with chronic functional constipation.^[35]^ A review conducted by Li et al on both animals and humans revealed the ability of acupuncture to regulate gastrointestinal function. The review showed that the acupoints ST36 (Zusanli), PC6 (Neiguan), and LI4 (Hegu) have the ability to increase or decrease gastric and colonic motility. The motility changes appear to be regulated depending on the patient’s condition. The review also found that the regulating effect of the acupoint ST36 (Zusanli) is often occurring through the vagal pathway, in cases where the ST36 (Zusanli) decreased colonic motility, it was done through Oxytocin expression.^[36]^ The array of evidence justifies the use of acupuncture for digestion-related conditions. Our study includes the acupoints ST36 (Zusanli), ST37 (Shangjuxu), ST39 (Xiajuxu), PC6 (Neiguan), and LI4 (Hegu) that were mentioned in the above studies, and in our intervention group acupuncture protocol.

In general, Acupuncture in cancer patients is considered a supplementary treatment that can contribute to the patient’s overall recovery, and reduce chemotherapy and radiation therapy side effects, but not a replacement of Western medicine therapies. A small meta-analysis on the efficacy of acupuncture in chemotherapy-induced peripheral neuropathy conducted by Chien et al^[37]^ found an effective ability of acupuncture to reduce peripheral neuropathy pain and functional limitation. Zhang et al^[38]^ conducted a meta-analysis including 10 studies on cancer-related fatigue. The meta-analysis found acupuncture to be an effective treatment for cancer-related fatigue. Jang et al^[39]^ found similar effects in an updated systematic review and meta-analysis on cancer-related fatigue for cancer survivors. Chiu et al^[40]^ in a systematic review and meta-analysis on cancer-related pain that included 29 studies found that acupuncture is effective in malignancy and surgical related analgesia and recommended that acupuncture can be combined with routine treatments for analgesia in cancer patients. A small meta-analysis found that acupuncture is also a beneficial treatment for cancer patients suffering from hiccups, however, the level of evidence was low.^[41]^ Ni et al^[42]^ conducted a meta-analysis on acupuncture for radiation-induced xerostomia and found some level of effectiveness in reducing xerostomia following acupuncture, although a low level of evidence.

Specifically in esophagus cancer patients, acupuncture was investigated in a study conducted by Tang et al on the application of acupuncture anesthesia in operation for carcinoma of the esophagus found a good analgesic effect during the surgery following acupuncture.^[43]^ Li et al^[44]^ used porous nano-sensing acupuncture for anesthesia in 30 esophageal cancer after one-lung ventilation thoracotomy and found a significant improvement in cognitive function, reduced level of inflammatory factors, and improved therapeutic effect. A small systematic review on acupuncture effect for radiation-induced xerostomia in patients suffering from head and neck cancer did not find any superior effect of acupuncture over the control but showed good safety for acupuncture on head and neck cancer patients.^[45]^ The effect of acupuncture on the esophageal organ in non-cancer patients is demonstrated in a study on the acupoint ST36 (Zusanli) that was shown to significantly reduce the lower esophageal sphincter resting pressure.^[46]^ In a case report, acupuncture was effective for oropharyngeal dysphagia and esophageal motility disorder.^[47]^ Overall acupuncture’s effect on esophageal cancer patients was not well investigated and more research on this intervention is needed.

This research will investigate a possible cooperation and integration approach of both Western and Chinese medicine in esophagus cancer treatment. In this integration, each treatment method focuses on its strength, western medicine in tumor treatment, and Chinese medicine in digestion symptoms in order to improve the patient’s condition and quality of life. This research will shed more light on the effectiveness of acupuncture as an intervention for both esophagus cancer patients and dumping syndrome. In the case that the study findings will be in favor of acupuncture, it will provide a valuable, low-cost supportive therapeutic option for advanced esophagus cancer patients with dumping syndrome.

Limitations of the study include a specific Taiwanese population and cannot be generalized to other populations. This study is also only a single-center study. The study protocol could not apply a double-blind design due to the type of intervention (manual acupuncture). The study will investigate the role of acupuncture in the prevention of dumping syndrome and malnourishment, the study does not require a positive Sigstad’s score to verify dumping syndrome as a condition for inclusion, therefore it is possible that the number of patients that will develop dumping syndrome could be low, in this case, the study will focus on body weight and BMI as the main outcomes to assess the prevention of malnourishment. The study did not specify the cancer stage in the inclusion criteria. High mortality rates in Esophagus cancer might impair the completeness of the data and can increase the levels of missing data.

## Author contributions

**Conceptualization:** Peter Karl Mayer, Pei-Yu Kao, Eyal Ben-Arie.

**Methodology:** Peter Karl Mayer, Pei-Yu Kao, Yu-Chen Lee, Yi-Fang Liao, Wen-Chao Ho, Eyal Ben-Arie.

**Resources:** Pei-Yu Kao, Yu-Chen Lee.

**Supervision:** Peter Karl Mayer, Pei-Yu Kao, Yu-Chen Lee, Wen-Chao Ho.

**Writing – original draft:** Peter Karl Mayer, Pei-Yu Kao, Eyal Ben-Arie.

**Writing – review & editing:** Peter Karl Mayer, Pei-Yu Kao, Yu-Chen Lee, Yi-Fang Liao, Wen-Chao Ho, Eyal Ben-Arie.

## Supplementary Material




