# Supplementary material for: Acupuncture effect on dumping syndrome in esophagus cancer patients with feeding jejunostomy: A study protocol for a single blind randomized control trial
Source: Medicine (Baltimore). 2023 Jun 9;102(23):e33895. doi: 10.1097/MD.0000000000033895 (PMC10256332; doi:10.1097/MD.0000000000033895)
Supplement: Supplementary file 1 [file medi-102-e33895-s001.pdf]

Supplementary Figure 1: The Sigstad's score

Shock +5
Fainting (syncope), unconsciousness +4
Desire to lie or sit down +4
Breathlessness (dyspnoea) +3
Weakness, exhaustion +3
Sleepiness, drowsiness, apathy, falling asleep +3
Palpitation +3
Restlessness +2
Dizziness +2
Headaches +1
Feeling of warmth, sweating, pallor, clammy skin +1
Nausea +1
Abdominal fullness, meteorism +1
Borborygmus +1
Eructation −1
Vomiting −4
