# Supplementary material for: Acupuncture effect on dumping syndrome in esophagus cancer patients with feeding jejunostomy: A study protocol for a single blind randomized control trial
Source: Medicine (Baltimore). 2023 Jun 9;102(23):e33895. doi: 10.1097/MD.0000000000033895 (PMC10256332; doi:10.1097/MD.0000000000033895)
Supplement: Supplementary file 2 [file medi-102-e33895-s002.pdf]

Supplementary Figure 2: the Arts' dumping questionnaire

**Early dumping syndrome symptoms**
Sweating
Flushing
Dizziness
Palpitations
Abdominal pain
Diarrhoea
Bloating
Nausea
**Late dumping syndrome symptoms**
Sweating
Palpitations
Hunger
Drowsiness and/or unconsciousness
Tremor
Irritability

1. Scarpellini E, Arts J, Karamanolis G, et al. International consensus on the diagnosis and management of dumping syndrome. *Nat Rev Endocrinol.* 2020;16(8):448-466.
